# Can a single question about family members’ sense of security during palliative care predict their well-being during bereavement? A longitudinal study during ongoing care and one year after the patient’s death

**DOI:** 10.1186/s12904-019-0446-1

**Published:** 2019-07-25

**Authors:** Anna Milberg, Maria Liljeroos, Barbro Krevers

**Affiliations:** 1 Department of Medical and Health Sciences, Linköping University, Linköping, Sweden; 2 Department of Advanced Home Care and Department of Medical and Health Sciences, Linköping University, Norrköping, Sweden; 3 Centre for Clinical Research Sörmland, Uppsala University, Eskilstuna, Sweden; 4 Medical Department, Mälarsjukhuset Hospital, 631 88 Eskilstuna, Sweden

**Keywords:** Family members, Palliative care, Security, Bereavement

## Abstract

**Background:**

It has been recognised that more evidence about important aspects of family members’ sense of security during palliative care is needed. The objectives of the study was: i) to discover what variables are associated with family members feeling secure during palliative care; ii) to develop a model of family members’ sense of security during palliative care, and iii) to evaluate if family members’ sense of security during ongoing palliative care predicts well-being during bereavement.

**Methods:**

Between September 2009 and October 2010, 227 family members (of patients admitted to six Swedish palliative home care units) participated in the study (participation rate 75%) during ongoing care and 158 participated also 1 year after the patient’s death (70%). They answered a single question regarding the family members’ sense of security during the palliative care period. The question was constructed and validated by the researchers. Data were also collected using other questions and validated instruments and analysed stepwise with Generalized Linear Models (ordinal multinomial distribution and logit link).

**Results:**

Sixteen variables were positively related to family members’ sense of security during ongoing palliative care. The five variables with the highest importance were selected into the model (listed in decreasing importance): Family members’ mastery; nervousness and stress; self-efficacy; patient having gynaecological cancer; family members’ perceived quality of life. Moreover, the family members’ sense of security during ongoing palliative care predicted ten variables indicating their well-being 1 year after the patient’s death, e.g. psychological well-being, complicated grief symptoms, health related quality of life.

**Conclusions:**

The findings reveal possibilities to identify family members at risk of negative adjustment to bereavement in clinical practice and may help to develop interventions to support family members during ongoing palliative care.

**Electronic supplementary material:**

The online version of this article (10.1186/s12904-019-0446-1) contains supplementary material, which is available to authorized users.

## Background

Life-threatening illness often generates considerable distress for family members [1], and having a sense of security can be of great value for them in such situations [2–6]. A definition of security is ‘freedom from danger’, ‘freedom from fear or anxiety’, and ‘safety’. To feel security in care is of international relevance. Funk et al. [7] found that a conceptualization of security extends beyond trust in individuals to include a generalized sense of institutional trust in the health care system. For family caregivers’, it provides security to know that healthcare will be provided when needed by competent professionals and it is of crucial importance, particularly for those, such as palliative family caregivers, that are already in situations that create a fundamental sense of insecurity.

Research has indicated that family members’ sense of security can be facilitated in palliative care, e.g. by the acquisition of adequate knowledge about palliative care and possible symptom management, by ensuring the availability of competent professionals with an attitude of open-mindedness, open-heartedness, and a team sensitivity and flexibility in meeting both patients’ and families’ needs [3, 5–11]. In addition, the importance of family caregivers feeling secure in their own identity and self-worth as caregivers and individuals have also been stressed [12].

Although family members’ sense of security during palliative care has been recognised as valuable and research suggesting that such a sense may be facilitated by professionals in palliative care, we have only identified one published study applying a quantitative design and with focus on variables associated with family members being secure/less secure. Igarashi et al. examined associations between end-of-life care in Japan and a sense of security regarding regional cancer care among bereaved families. Variables significantly associated with a higher sense of security in family members of patients that had died due to cancer were the family members’ higher age, patient’s death at home, better health status of the family at patients’ end of life, lower caregiving burden, and elements of perceived good patient death, including being free from physical distress, trusting the physician, living in calm circumstances, and feeling that one’s life was fulfilling [11].

Hence, having a sense of security can be of great value for the family member during palliative care, but few studies have focused on variables associated with family members being secure/less secure and previous quantitative research has had a post-loss, cross-sectional design. To further develop palliative care and the support given to family members, it seems valuable to further investigate what variables are related to family members feeling secure or insecure during ongoing palliative care and to study other contexts than the Japanese one, to assist staff members to identify individuals at risk and plan effective support. In addition, to our knowledge, there is no study exploring longitudinal data regarding family members’ sense of security. Therefore, we wanted to study what characterises family members feeling secure-insecure during palliative care in a Swedish context, and also to evaluate if there is an association between their sense of security during ongoing care and well-being during bereavement.

### Aims

The aims of the present analyses were: 1) to discover which variables are associated with family members feeling secure during the palliative care period; 2) to develop a model of family members’ sense of security during palliative care, and 3) to evaluate if family members’ sense of security during ongoing palliative care predicts well-being during bereavement.

### Main hypotheses

We hypothesised that the family members’ sense of security in palliative care would be related to the family members’ demographic characteristics, health-related quality of life, coping, attachment security, perceived situation as a family member of a severely ill person, perceived support from family and friends, sense of security during palliative care and patient characteristics, and inversely related to the family members’ perceived stress. These hypotheses were based on previous findings in the literature suggesting that family members’ sense of security in palliative care or advanced cancer care is associated with family member demographics, e.g. older age [11], family members’ health-related quality of life, e.g. better health status [11], situation as family member of a severely ill person, e.g. better caregiving situation [11]. We also hypothesised that family members’ perception of stress and coping, their attachment security, sense of security with care, patient characteristics, and perceived support from family and friends would be associated with family members’ sense of security during palliative home care, because such aspects have been found important in relation to patients’ sense of security during palliative care [13] or when they have advanced cancer [14], or the general population’s feelings of security regarding cancer care [15].

In the longitudinal analysis, we hypothesised that the family members’ sense of security in palliative care would be related to the family members’ well-being during bereavement in terms of their health-related quality of life, stress and coping, psychological well-being and complicated grief symptoms. The reason for these hypotheses was that previous longitudinal studies of bereaved family members have indicated that well-being post-loss can be assessed in terms of health-related quality of life [16], psychological well-being [15, 16], stress [17, 18], and complicated grief symptoms [19].

## Methods

### Design

This questionnaire study had two different types of data collection: i) data collected during ongoing palliative care (which were used in the cross-sectional analyses related to aim 1–2); ii) data collected during bereavement (which were used together with the data collected during ongoing palliative care in the predictive analyses related to aim 3).

### Participants, procedure and measures

Details of the data collection have already been published and is briefly summaries here [13, 20]. Family members of patients who were receiving palliative home care were recruited from six palliative home care units in Sweden. The cross-sectional analyses were based on a sample of 227 participants, and the bereavement analyses were based on 158 participants, the demographic characteristics are displayed in Table 1. The data during ongoing palliative care were collected between September 2009 and October 2010, and data 1 year into bereavement were collected 1 year later.

**Table 1:**
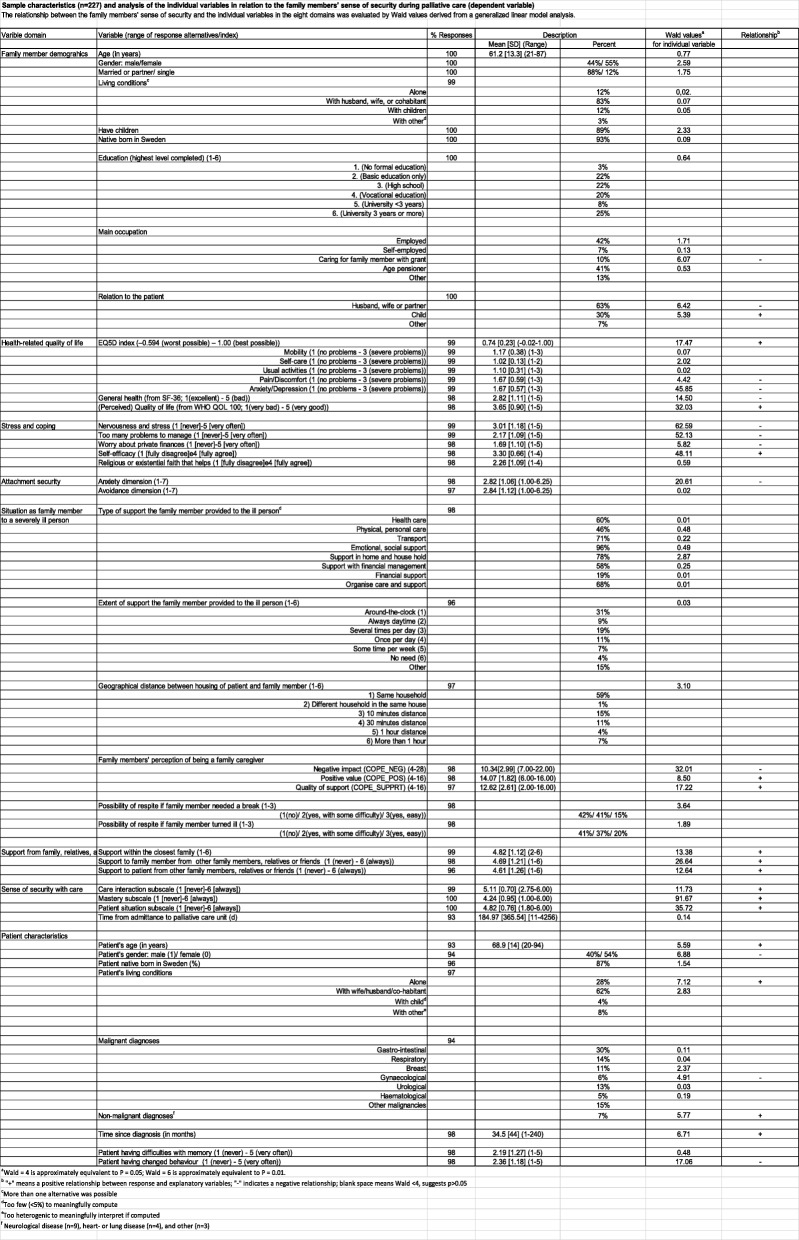
Sample characteristics (*n* = 227) and analysis of the individual variables in relation to the family members’ sense of security during palliative care (dependent variable)

Consent from the participants were collected in three steps:

1. Eligible family members (according to inclusion and exclusion criteria) received oral and written information and was asked (by the health care staff) if they wanted to participate, and to reply by filling in a form (written informed consent). The form was (according to the family member’s choice) either returned to the research nurse by the health care staff or posted by the family member to the research nurse in a prepaid envelope.
2. The research nurse then contacted these family members who had provided written informed consent by phone. The research nurse gave more information about the study and checked that the family member still wanted to participate. A date for the interview was also scheduled at the call. (99% of the family members wanted to be interviewed over the phone).
3. Before the interview started (at the set date) the research nurse repeated information about the study, including the right to withdraw at any time without giving any reason, and she also checked that the family member still wanted to participate.

In the form giving informed consent to participate in the study (see Step 1 above), there was also another option of receiving more information from a research nurse before deciding. Those family members that were interested in getting more such information (a minority) were then contacted by phone by the research nurse. She checked if the family member was still interested to get more information. After providing such information, the research nurse asked if the family member wanted to participate, and a date was scheduled for the interview (i.e. Step 2). The family members who then decided to participate were the family members who provided verbal consent only.

Before the actual interview with the participants providing verbal consent only, the research nurse went through Step 3 (see above), that is she checked at the set date, before the interview, that the family member had not changed their mind about participation.

The reason for not asking the family members who provided verbal consent only to send a written informed consent, was to avoid burdening them with an administrative task in the difficult situation of having a severely ill and dying close relative or friend.

The issue that was the focus of this study (i.e. the dependent variable), family members’ sense of security during the palliative care period, was assessed by one question constructed by the authors (“How often have you felt secure [during the palliative care period]?”; six-point response-scale, 1 (never) to 6 (always)), and initial analysis regarding construct validity was conducted [20]. We also constructed a question where we asked about how important it was for the family member to sense security with the care of the ill family member (“How important is it for you to sense security with the care of your member?”; four-point Likert scale 1 (Of no importance) to 4 (Of the utmost importance). Regarding content and face validity of these questions, both were considered as valid in relation to the aim of the study by an expert panel of researchers (*n* = 3) and clinicians (*n* = 6).

A pilot study was conducted with four relatives (one during home visits and three through telephone interviews) and they also were asked about the questionnaire and the method of collecting data. This led to corrections to the instructions and minor revisions to some of the questions, but not to the two questions above mentioned.

When we selected questions and instruments, we wanted to be mindful of the family members’ situations in having a close family member (i.e. the patient) who was dying or had died during the last year, and therefore short scales or single questions were chosen if feasible. For further information about the measures, see Table 2 and Additional file 1. Most of the data was gathered by a structured interview with the family member (ongoing care and 1 year after the patient’s death) using questionnaires that were administered verbally, and some data were collected via the palliative care team, for example the patients’ diagnoses.

**Table 2:** Overview of measured variables collected during on-going palliative care and one year after the patient’s death

Variable domain	Measures	Origin
Family member demographics	Age, gender, living and family conditions, education, country of birth, relationship to the patient, and main occupation	*
Health-related quality of life	The EuroQol-5D (EQ-5D), including five subscales: mobility, self-care, pain, usual activities, and psychological status; 3-point response scale: 1 (no problems) - 3 (severe problems).	
	An index score was calculated for each respondent’s health status: 1 = full health; − 0.594 = worst-imaginable health state	[21]
	General quality of life: one question (of 100) from the WHO QOL 100 instrument; 5-point scale: 1 (very poor) – 5 (very good)	[22]
	General health: one overall question from the SF-36 (a 36-item short-form health survey questionnaire); 5-point scale: 1 (excellent health) – 5 (poor health)	[23]
Stress and coping	Stress: two (of ten) items from the Perceived Stress Scale (PSS) (felt nervous and “stressed”; difficulties were piling up so high that you could not overcome them); 5-point scale: 0 (never) – 4 (very often)	[24]
	Worry about personal finances during the last month: 5-point scale: 0 (never) – 4 (very often)	*
	Self-efficacy: One statement (of ten; I can solve most problems if I invest the necessary effort) from the General Self-Efficacy Scale (GSE); 4-point scale: 1 (not at all true) – 4 (exactly true)	[25, 26]
	Religious or existential belief that helps the informant to cope with problems: One statement; 4-point scale: 1 (not at all true) – 4 (exactly true)	*
Attachment security	the Experiences in Close Relationships scale (ECR-M16); 16 items to measure attachment anxiety (fear of rejection and abandonment) and avoidance (discomfort with closeness and dependence on close others) in close relationships (including non-romantic partners); 7-point scale: 1 (lower attachment insecurity) - 7 (greater attachment insecurity)	[27]
Situation as family member to a severely ill person	Type of support/care the family member provided to the ill person: eight alternatives; yes/no (see Table 1)	*
	Extent of support the family member provided to the ill person: one question; 6-point scale: 1 (around-the-clock) – 6 (no need of support)	*
	Geographical distance between housing of patient and family member: one question; 6-point scale: 1 (same household) - 6 (more than 1-h distance)	*
	The family member’s perception of being a family caregiver: the COPE questionnaire: 15 questions; 4 point scale: 1 (never) – 4 (always) based on 3 validated sub-scales: Negative impact scale, Positive value scale and Quality of support scale	[28]
	Possibility of respite if family member needed a break: one question; 3-point scale: 1 (no) – 2 (yes, with some difficulty) – 3 (yes, easy)	*
	Possibility of respite if family member became ill: one question; 3-point scale: 1 (no) – 2 (yes, with some difficulty) – 3 (yes, easy)	*
Support from family, relatives, and friends	The family member’s perception of support from members within the closest family: one question; 6-pointscale: 1(never) – 6 (always)	*
	The family member’s perception of support from other family members, relatives or friends: one question; 6-pointscale: 1(never) – 6 (always)	*
	The family member’s perception of support to patient from other family members, relatives or friends: one question; 6-pointscale: (1 (never) - 6 (always))	*
Sense of security in palliative care	The family members’ sense of security with palliative care: The sense of security in care-Relatives’ Evaluation instrument (15-item instrument (6-point scale: 1 (never) - 6 (always) based on 3 validated sub-scales: Care Interaction (eight items), Mastery (four items) and Patient Situation (three items)	[20]
	Time (days) from commencement of palliative home care services () to the interview (with the family member)	Medical records
Patient characteristics	Demographics: age, gender, living and family conditions, and country of birth.	*
	Diagnosis: the patient’s diagnosis and the time since diagnosis.	Medical records
	(Family member’s perception of) Patient having difficulties with memory: one questions; 5-point response scale: 1 (never) – 5 (very often)	*
	(Family member’s perception of) Patient having changed behavior: one questions; 5-point response scale: 1 (never) – 5 (very often)	*
Well-being during bereavement	The EuroQol-5D (EQ-5D), see above “Health-related quality of life” domain	[21]
	General quality of life, one question from the WHO QOL 100 instrument, see above	[22]
	General health: one overall question from the SF-36, see above “Health-related quality of life” domain	[23]
	Psychological well-being previous 2 weeks: The WHO-5 Well-being Index 6–30 (five items; 6-point scale: 1(‘at no time’) – 6 (‘all of the time’))	[29]
	Stress: two (of ten) items from the Perceived Stress Scale (PSS); see above “Stress and coping” domain	[24]
	Had own contact with healthcare because of the death of the patient (that received care by the palliative care unit); 4-point scale: 1(never) - 4(> 5 times)	*
	Complicated grief symptoms: Inventory of complicated grief screen (ICGS) is a 9-item shortened version of the original ICG; 5-point scale: 1(never) - 5(always)	[30, 31]

*Questionnaire developed by the authors

### Statistical analyses

An assessment of missing data did not indicate any systematic patterns, and the number of missing values was small, (0% (e.g. on the dependent variable, i.e. the Family members’ feeling of security during the palliative care period) - 6% (13/227; Patient’s age (in years)). Where there were missing values, the specific analysis was performed with this respondent’s information excluded, although the respondent could be included in other analyses.

Because of the substantial number of independent variables in the cross-sectional analyses and the possibility of over-fitting the model, the variables that were hypothetically important to the family members’ sense of security during ongoing palliative care (the dependent variable) were gathered into eight domains. The domains were then managed as steps in the analyses, lowering the total of variables tested in one particular test. The eight domains with possible association to family members’ sense of security during ongoing palliative care were assessed by the variables listed in Table 2. This table also shows the variables that were collected as indicators of family members’ well-being during bereavement and of hypothesized predictors.

Descriptive statistics were conducted for the individual variables in the eight domains. The relationship between family members’ sense of security and the independent variables in the eight domains was evaluated by Wald values derived from the generalised linear model analysis with ordinal multinomial distribution and logit link or normal identity. In this type of model, the dependent variable is treated as an ordinal multinomial variable (as opposed to categorical or linear/continuous variable), which best reflected the nature of the dependent variable in our response data (i.e. a six-point response-scale).

Wald = 4 is approximately equivalent to *P* = 0.05, and Wald = 6 is approximately equivalent to *P* = 0.01. The Akaike Information Criterion (AIC) was used in the model-building analyses [32]. The AIC offers a relative measure of the information lost when a given model is applied to illustrate reality. The simplest best model with the smallest information loss when predicting the outcome gives the lowest AIC value.

As the first step (Step 0) in the model-building process, one analysis per domain was carried out, and its AIC was used to determine in which sequence the domains should be added in the subsequent stepwise procedure. Only variables with Wald values > 2 were allowed to be tried in the further model-building. This Wald value corresponds to approximately *p* < 0.15; hence, a generous selection criterion was preferred, to reduce the risk of discarding variables that could have been valuable in further model-building. The domain with the lowest AIC was taken first in the model-building analyses and the domain with the highest AIC was taken last, because a lower AIC value signals a higher value of explanation. The final step in the process was performed using the best subset analyses with AIC. Classification of the developed model was calculated and the percentage of correct classifications of the observed cases was computed. In addition, we made statistical comparisons between the family members who took part and those who declined to participate (chi-square and t-test). Statistical tests were two-tailed with alpha set at 0.05. The data were analysed using Statistica, Version 10 (Statsoft Inc., USA).

## Results

The respondents’ ratings of their sense of security during palliative care ranged from 1 (never) to 6 (always), with a mean value of 4.11 (standard deviation (SD) 1.27). When asked about how important it was for the family member to sense security with the care of the (ill) family member, i.e. the patient receiving palliative home care, the ratings ranged from 3 (Of great importance) to 4 (Of the utmost importance), with a mean value of 3.9 (SD 0.31).

### Aim 1: family members’ sense of security during ongoing palliative care

According to the analysis of the individual variables, all hypothesised domains were significantly related to the dependent variable, i.e. to the family members’ sense of security during palliative care.

The following 16 variables were positively related to family members’ sense of security during ongoing palliative care (presented in order of decreasing Wald values; 91.67–5.39): Mastery subscale (SEC-R); Self-efficacy; Patient Situation subscale (SEC-R); (Perceived) Quality of life; (Perceived) Support to family member from other family members, relatives or friends; EQ5D index; Quality of support (COPE_SUPPORT); (Perceived) Support within the closest family; (Perceived) Support to patient from other family members, relatives or friends; Care interaction subscale (SEC-R); Positive value of being a family caregiver (COPE_POS); Patient living alone; Time since diagnosis; Patient having a non-malignant diagnosis; Patient’s age; and Family member being a child to the patient.

The following 13 variables were negatively related to family members’ sense of security during ongoing palliative care (presented in order of decreasing Wald values; 62.59–4.42): Family member being nervous and feeling stress; Family member perceiving too many problems to manage; Anxiety/Depression subscale; Negative perception of the impact of being a family caregiver (COPE_NEG); Attachment anxiety; Patient having changed behaviour; Family member’s general health; Patient being male; Family member being husband, wife or partner to the patient; Family member caring for the patient with grant; Family member’s worry about private finances; Patient having gynaecological cancer; and Family member’s pain/discomfort.

### Aim 2: model-building

Model-building began with one analysis per domain. Only the variables with Wald > 2 were chosen for further analyses in the steps. The AIC for the eight domains resulted in “Sense of security with care” domain was entered first in the stepwise procedure, and “Family member demographics” domain last.

The stepwise model-building process resulted in a model with the following five variables (presented in order of decreasing Wald values): i) Mastery subscale 48.44; 2) Nervousness and stress 18.84; 3) Self-efficacy 13.32; 4) (Patient having) Gynaecological cancer 9.46; 5) (Perceived) Quality of life 3.40 (Table 3).

**Table 3:** Final model in the stepwise model-building process

Varible domain	Variable	Wald values^a^ for partial regression coefficients in the final best subset	Relationship^b^
Sense of security with care	Mastery subscale (SEC-R; 1 [never] - 6 [always])	48.44	+
Stress and coping	Nervousness and stress (from PSS; 1 [never] - 5 [very often])	18.84	–
	Self-efficacy (from GSE; 1 [fully disagree] - 4 [fully agree])	13.32	+
Patient characteristics	Gynecological cancer	9.46	–
Health-related quality of life	Quality of life (from WHO QOL 100; 1 (very bad) - 5 (very good))	3.40	+

A model was developed with the family members’ sense of security during ongoing palliative care as the dependent variable, and the variables in the eight domains as independent variables

^a^Wald = 4 is approximately equivalent to *P* = 0.05; Wald = 6 is approximately equivalent to *P* = 0.01

^b^“+” means a positive relationship between response and explanatory variables; “-” indicates a negative relationship

Of the 209 respondents used in the selected model, the correct response alternatives were predicted in 55%, with a range from the individual response alternatives from 0% (1 (never) as 1 (never) to 60% (4 (often) as 4 (often)). A correct response plus minus one response alternative (e.g., response alternative 5 (very often) as 4 (often), 5 (very often) or 6 (always)) was predicted in 91%. It seems important to avoid the risk of overestimating a family member’s sense of security; therefore, a correct response minus 1 response alternative (e.g., response alternative 5 (very often) as either 4 (often) or 5 (very often) was calculated: 68%.

### Aim 3: family members’ sense of security during ongoing palliative care as a predictor of well-being during bereavement

The family members’ sense of security during ongoing palliative care predicted ten variables collected 1 year after the patient’s death (see Table 4). Three of these variables were positively related to the family members’ sense of security during ongoing palliative care (presented in order of decreasing Wald values; 23.75–5.74): Psychological well-being; (Perceived) Quality of life; Health-related quality of life.

**Table 4:** Characteristics and longitudinal analysis of the individual variables’ relation to the family members’ sense of security during ongoing palliative care (independent variable)

Varible domain	Variable (range of response alternatives/index) *n* = 158	% Responses	Description Mean [SD] (Range) or Percent	Wald values^a^	Relation-ship^b^
Wellbeing during bereavement	Health-related quality of life: EQ5D index (−0.594 (worst possible) – 1.00 (best possible))	100	0.78 (0.23 [−0.041–1.00])	5.74	+
	Mobility (1 (no problems) - 3 (severe problems))	100	1.21 (0.41 [1–2])	0.60	
	Self-care (1 (no problems) - 3 (severe problems))	100	1.02 (0.14 [1–2])	2.00	
	Usual activities (1 (no problems) - 3 (severe problems))	100	1.08 (0–30 [1–3])	0.01	
	Pain/Discomfort (1 (no problems) - 3 (severe problems))	100	1.63 (0.60 [1–3])	3.91	–
	Anxiety/Depression (1 (no problems) - 3 (severe problems))	100	1.47 (0.55 [1–3])	17.60	–
	General health (from SF-36; 1 (excellent) - 5 (bad))	100	2.85 (1.11 [1–5])	7.45	–
	Quality of life (from WHO QOL 100; 1 (very bad) - 5 (very good))	100	4.03 (0.89 [1–5 9)	14.95	+
	Nervousness and stress (from Perceived Stress Scale; 1 (never) -5 (very often))	100	2.48 (1.21] (1–5)	17.26	–
	Too many problems to manage (from General Self-Efficacy Scale; 1 (never) -5 (very often))	99	1.64 (0.97 [1–5])	5.94	–
	Psychological well-being (WHO-wellbeing index; 6–30; 1 (at no time) -6 (all of the time))	100	20.87 (5.83 [6–30])	23.75	+
	Had own contact with health care because of the death of the patient	99	1.49 [0.91] (1–4)	12.23	–
	Complicated grief symptoms (Inventory of complicated grief screen; 1 (never) -5 (always))	100	2.41 (0.77 (1–4.56])	23.55	–

The relationships between the family members’ sense of security and the individual variables were evaluated by Wald values derived from a generalized linear model analysis

^a^Wald = 4 is approximately equivalent to *P* = 0.05; Wald = 6 is approximately equivalent to *P* = 0.01

^b^ “+” means a positive relationship between response and explanatory variables; “-” indicates a negative relationship

The following seven variables were negatively related to family members’ sense of security during ongoing palliative care (presented in order of decreasing Wald values; 23.55–3.91): Complicated grief symptoms; Anxiety/Depression; Nervousness and stress; Contact with health care post-loss because of the death of the patient; General health; Too many problems to manage; Pain/Discomfort.

## Discussion

This study showed that the family members rated their sense of security during palliative care rather high (mean 4.11 (Likert-type score 1–6), and they perceived a sense of security during ongoing palliative care of great or utmost importance (mean 3.9 of maximum 4 (=utmost)). All the hypothesised domains (i.e. the family members’ demographic characteristics, health-related quality of life, stress, coping, attachment security, perceived situation as a family member of a severely ill person, perceived support from family and friends, sense of security during palliative care and patient characteristics) were significantly related to the family members’ sense of security during the palliative home care period, and we developed a (cross-sectional) model for prediction of such a sense. In addition, the longitudinal analysis showed that the family members’ well-being 1 year after the patient’s death was predicted by their sense of security during ongoing palliative care.

The developed model pointed out that family members sensing less security during ongoing palliative care also rated lower mastery, higher nervousness and stress, lower self-efficacy and lower health-related quality of life. These results have support in the previous literature [11, 13–15], although family members’ ages were not associated with their sense of security, as in Igarachi et al.’s study [12].

As the same, or very similar, questions were posed to patients in a parallel study (with focus on patients’ sense of security during palliative care) [13] and were analysed with similar methods, it is worthwhile to point out the variables that were selected in both FC and patient models: perception of nervousness and stress; self-efficacy; and patients having gynaecological cancer. The patient model also included “worrying about personal finances”, and “avoidance”, while the FC model comprised FC mastery and FC perceived quality of life as well. Although patients and FC seem to share several aspects related to their sense of security during palliative care, there are also differences to be recognised by the health care staff.

Moreover, the findings indicate that the content of a single question on the family members’ sense of security is of significance to their situation and of relevance for palliative care. The Mastery subscale had the highest Wald value of the selected variables in the model. This subscale, one of three in the Sense of Security in Care - Relatives’ Evaluation (SEC-R) instrument [20], includes questions on e.g. the family member’s feelings of control, of ability to be oneself when interacting with the patient’s health care personnel and of ability to do what is most important in daily life given the patient’s health and care. In clinical practice, attention and support to such dimensions may facilitate the family member’s sense of security, and when appropriate it seems of value to initiate a dialogue with the family member about such issues.

The final model also included a fifth variable, namely the patient having gynaecological malignancy. However, there were only 13 family members in the study population whose ill family member (the patient) had such a diagnosis. These results should, therefore, be interpreted with caution, and the importance of gynaecological malignancy on family members’ sense of security during palliative care needs further study.

Four of the hypothesised domains - Family members demographics; Attachment (in)security; Situation as family member to a severely ill person; (Lack of) Support from family, relatives and friends - were not represented in the final model, but showed significant associations with the family members’ sense of security in the bivariate analysis. These findings also seem of importance when clinicians want to identify family members at risk of sensing low security during ongoing palliative care.

Although the mean value of the family members’ ratings of their sense of security during palliative care was rather high (as pointed out above), it is interesting that the mean value seems somewhat lower than when patients during ongoing palliative care responded to the same question (4.6 (SD 1.19)) [13] . These results are in line with previous research showing higher anxiety levels in family members compared to patients in palliative care [33, 34].

Finally, we want to draw attention to the results of the longitudinal analysis suggesting that the family members’ well-being (e.g. in terms of psychological well-being, complicated grief symptoms, quality of life) 1 year after the patient’s death was predicted by their sense of security during ongoing palliative care. Previous studies have identified risk factors for negative adjustment to bereavement (e.g. a closer relationship to the deceased, socioeconomic disadvantage) as well as positive factors for reduced risk (e.g. positive high-quality social support, caregiver report of needs met) [35–39]. Moreover, instruments to identify family members at risk for developing bereavement-related mental health challenges pre- or post-loss have been developed (e.g. Family Relationships Index (FRI) [40] and the Bereavement Risk Inventory and Screening Questionnaire (BRISQ)) [37], and pre-loss screening as well as interventions initiated pre-loss have been advocated to facilitate caregivers’ return to normal life as soon and as effectively as possible [41, 42]. To our knowledge, no study with a quantitative design has previously focused on sense of security as a possible pre-loss resilient factor during the delivery of the patient care. According to the present findings, family members’ sense of security while the patient is still alive should be further studied to clarify its role with regard to such outcomes and possible future systematic implementation in clinical practice.

### Limitations

One part of the study had a cross-sectional design, and, thus, the observed associations among these variables collected during ongoing palliative care may not be causal. Moreover, the mean value of the family members’ sense of security scores was rather high, which may limit the conclusion that can be drawn about populations with low scores on this measure. In addition, 93% of the responders were born in Sweden. Research is needed to examine the sense of security during palliative care especially among vulnerable groups of family members, including those with limited ability to communicate verbally with health care staff.

Also, data is collected 8 years back in time. During that time period there has not been any large changes regarding the organisation of the palliative care, possibly it has expanded to patients with non-malignant diseases, and the length of stay in hospital has become shorter since the data were collected. This makes it more important to recognise patients and family members needs in the palliative homecare context.

Finally, single questions may contribute with limitations of the study. Although most of these questions were selected from valid and reliable instruments, a few were constructed by the authors, including the question about family members’ sense of security. As psychological phenomena are usually not directly measurable, multiple item scales are often used to approximate a measure of the concept and to partly manage the effect of measurement error. However, at the time of the study there were, to our knowledge, no such multiple item scales to measure the study phenomenon. The single questions that were used were validated prior to the study concerning their content and face validity, but not statistically scrutinized concerning their construct and estimation of measurement error and this may have implications for the interpretations of the results.

## Conclusion

This study has identified several variables associated with family members having a sense of security during palliative care, and also developed a model for statistical prediction of such sense. Based on our findings, a single question about the family members’ sense of security during ongoing palliative care that predicted their well-being 1 year after the patients’ death may be a contribution to such development.

Moreover, future research should also explore effective interventions to support family members during ongoing palliative care as well as to prevent negative adjustment to bereavement. Although our study was not an intervention study, the factors identified as associated with the family members’ sense of security during ongoing care may inform the design of such future interventions.

## Additional file

Additional file 1: information Questionnaire developed by the authors. (DOCX 24 kb)

## Data Availability

Data origin from the “Upplevelse av trygghet hos patienter respektive anhöriga vid palliativ vård” study. Ethical restrictions prevent this data from being deposited in a public repository. The data are available upon request, and requests can be sent to the authors.

## Abbreviations

AIC: Akaike Information Criterion
ECR-M16: Experiences in Close Relationships scale
EQ-5D: The EuroQol-5D
GSE: General Self-Efficacy Scale
ICGS: Inventory of complicated grief screen
PSS: Perceived Stress Scale
SF-36: 36-item short-form health survey questionnaire
