# Supplementary material for: Can a single question about family members’ sense of security during palliative care predict their well-being during bereavement? A longitudinal study during ongoing care and one year after the patient’s death
Source: BMC Palliat Care. 2019 Jul 25;18:63. doi: 10.1186/s12904-019-0446-1 (PMC6657130; doi:10.1186/s12904-019-0446-1)
Supplement: Supplementary file 1 — information Questionnaire developed by the authors. (DOCX 24 kb) [file 12904_2019_446_MOESM1_ESM.docx]

**Additional file 1 information Questionnaire developed by the authors**

**Family member demographics:**

How old are you?

Male/female?

Living arrangements:

Do you; live alone, live with spouse, live with child, live with siblings, live with others?

If with others, with whom?

Do you have children? If so, how many? How many are living in the same household as you?

In which country are you born?

What is your highest level of education?

1. No formal education 2) Basic education only 3) High school 4) Vocational education 5) University <3 years 6) University 3 years or more

What is your relation to the patient?

1. Husband, wife or partner 2) Child 3) Other

What is your main occupation?

1. Employed 2) Self-employed 3) Caring for family member with grant 4) Age pensioner 5) Other

**Stress and coping**

How often have you been worry about personal finances during the last month?

1. Never
2. Rarely
3. Sometimes
4. Often
5. Very often

I have a religious belief or existential opinion that helps me to cope with my problems

1. Not at all true
2. Partly disagree
3. Partly agree
4. Exactly true

**Situation as family member to a severely ill person**

What type of support/care do you provide to X [the patient]? _______ eight alternatives; all yes/no

1. Help with health care e.g. help with medication or other treatment
2. Help with physical/personal care, e.g. hygiene, dressing, eating or toilet visits
3. Help with transport or movement inside or outside, to get to other places
4. Help with emotional/social support, for example, company, employment, encouragement
5. Help with homes and households, e.g. housework, cleaning, laundry
6. Help with financial management, e.g. ensure that bills are paid by relatives' money
7. Financial support for example your loved one gets money from you
8. Help with organizing and managing care and support e.g. contact care or home care services etc.
9. **Hur ofta får din närstående tillsyn, stöd eller hjälp av dig med anledning av hans/hennes sjukdom**

How often do you provide support/care to X [the patient]? one question; 6-point scale:

1. Around-the-clock
2. Always daytime
3. Several times/day
4. Once a day
5. Once a week
6. In no need of

How close do live to X [the patient]? one question; 6-point scale: 1 (same household) - 6 (more than 1-hour distance)

1. Living in the same household
2. Living in other household in the same building
3. I can go to the patient within 10 minutes
4. I can go to the patient within 30 minutes
5. I can go to the patient within one hour
6. It is more than 1-hour distance to get to the patient

Is there anyone else who could provide support to your relative in your place if you would need a break or temporary respite?

1. No, there is no one else
2. Yes, with some difficulty
3. Yes, easily

Is there anyone else who could give support to your relative in your place if you got sick?

1. No, there is no one else
2. Yes, with some difficulty
3. Yes, easily

**Support from family, relatives, and friends**

According to your experience, have members within the closest family supported X [the patient]?

1. Never
2. Rarely
3. Sometimes
4. Often
5. Almost always
6. Always

Have other family members within the closest family supported you?

1. Never
2. Rarely
3. Sometimes
4. Often
5. Almost always
6. Always

Have other family members, relatives or friends supported you?

1. Never
2. Rarely
3. Sometimes
4. Often
5. Almost always
6. Always

**Patient characteristics**

Do you perceive that X [the patient] have obvious difficulties with memory, e.g. to know what date it is or to recognize friends and neighbors?

1. Never
2. Rarely
3. Sometimes
4. Often
5. Very often

Do you perceive that X [the patient] have changed behaviour which make you sad or frustrated?

1. Never
2. Rarely
3. Sometimes
4. Often
5. Very often

**Well-being during bereavement**

Have you had own contact with health care because of the death of X [the patient (that received care by the palliative care unit)]; 4-point scale: 1(never) - 4(>5 times)
